# Roles of perception of similarities, continuum beliefs, and social distance toward a person with schizophrenia: a German sample study

**DOI:** 10.1007/s00127-023-02423-1

**Published:** 2023-01-28

**Authors:** V. Buckwitz, V. Juergensen, M. Göbel, G. Schomerus, S. Speerforck

**Affiliations:** 1 Department of Psychiatry, Medical Faculty, University of Leipzig, Semmelweisstr. 10, 04103 Leipzig, Germany; 2 Department of Psychology, University of California-Berkeley, Berkeley, USA

## Abstract

Continuum beliefs (CB) interventions aim to reduce the stigma of schizophrenia by stating that people with and without schizophrenia exist on one mental health-illness continuum. The current study examines the interrelation between perception of similarities (PoS), CB and desire for social distance (SD) in a German population sample (*N* = 760). Applying linear regression models: PoS and CB were significantly associated with lower SD explaining about 12% variance and PoS was significantly associated with higher CB. CB partially mediated the relation of PoS and SD. Upcoming mental illness stigma interventions applying CB interventions should also aim to increase PoS.

**Supplementary Information:**

The online version contains supplementary material available at 10.1007/s00127-023-02423-1.

## Introduction

Schizophrenia is one of the most stigmatized mental illnesses with severe consequences for those who are living with the condition [14]. Symptoms of schizophrenia are often perceived as far apart from typical behavior thus are people with schizophrenia—often placed in the social category of the “mentally ill”, presumed to be dangerous and unpredictable [1].

Social psychological research identified severe consequences for those who are perceived as outgroup members rather than ingroup members [8–10]. Influenced by empirical evidence from ingroup–outgroup theories, Link and Phelan’s [12] conceptualization of mental illness stigma described the process of stigmatization to result in the separation between “us vs. them”. Furthermore, Corrigan et al. [5] captured schizophrenia-related stigma within an empirical study as perceived differences between participants and a case description of a person with schizophrenia identifying the perception of differences as a key component in the process of stigmatization.

To diminish the notion of fundamental differentness between people with and without mental illness, continuum beliefs (CB) messages include assertations such as “Basically we are all sometimes like this person. It is just a question of how pronounced this state is.” [17]. Thereby, CB comprise assumptions that people with and without mental illness are similar and that forms of mental illness can vary on a continuum with typical behavior. Thereby, CB focus on similarities and attempt to overcome the implicit assumption of a given ingroup (e.g., “the mentally healthy”) vs. outgroup (e.g., “the mentally ill”) order.

To reveal the roles of perception of similarities (PoS) and CB in schizophrenia-related stigma reduction, this study is the first to address associations between PoS, CB, and desire for social distance (SD) toward a person with schizophrenia in a sample survey in Germany. First, increased PoS and higher CB are both expected to be associated with reduced SD. Second, increased PoS and higher CB are expected to be related to each other. Third, the stigma-reducing mechanism of PoS is hypothesized to be mediated by CB.

## Method

### Participants and procedure

A population online survey was carried out by USUMA, a social and market research company recruiting participants based on quota sampling (gender, age, and regional difference on state level) of the German population. Participants 18 + years old and proficient in the German language in Germany in July 2021 were addressed (see supplement table 1). Out of 1359 responses, 167 missed attention checks, 98 dropped out due to incompletion, 63 were screened out for rapid-guessing, and additional 271 dropped out due to failure to reply to the control question regarding the vignette or had missing data. Overall, 760 participants (53.55% female, 46.05% male, and 0.39% divers) were included in the study. After answering several sociodemographic questions, participants were asked to read a case description about a person described with symptoms of schizophrenia without using the term “schizophrenia” of about 230 words [2].

### Measures

Following the case description, individuals indicated their willingness to interact with the descripted person, using a 7-item social distancing scale [13]. Responses to a five-point Likert scale were then reversed, so that higher scores reflect a greater SD (*α* = 0.89, *M* = 3.49, SD = 0.85). Afterward, participants rated the degree to which they agreed with the following statement: ‘‘Basically we are all sometimes like this person. It’s just a question as to how pronounced this state is’’ on a five-point Likert scale [17]. Higher scores represented stronger CB about the existence of schizophrenia (*M* = 2.42, SD = 1.29). Finally, a German translation of Corrigan et al.’s [5] Semantic Differential: Similar-Different Scale was applied to capture perceived similarity to the person depictured in the vignette on a nine-point scale. A total score was determined by averaging responses across the three items, larger scores indicating greater perceived similarity (*α* = 0.89, *M* = 1.67, SD = 1.25). All scales showed good internal consistency.

### Data analytic plan

**Fig. 1:**
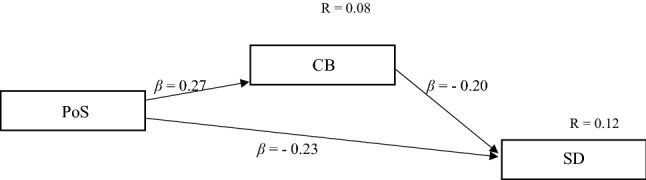
Mediation model of Continuum Beliefs (CB) and Perception of Similarities (PoS) predicting Desire for Social Distance (SD) Effect of mediation and all shown path coefficients are significant. Effect of mediation equals *β* = − 0.05, CI_95_ [− 0.08, − 0.03]

First, linear regression was applied to test the three hypothesized relations between (1) PoS and SD, (2) CB and SD, and (3) PoS and CB. Second, following Hayes and Scharkow [11], linear regression models with percentile bootstrap confidence intervals were used to test for a mediation effect of CB in the relation of PoS and SD (see supplementary table 2 and Fig. 1). Paired Bootstrapping (see [7] with 100.000 samples, each of the same size as the original sample (i.e., 760 individual observations), was used to estimate confidence intervals. The same bootstrapping procedure was applied for the other regression models to achieve robustness against mild deviations from normality of the residuals—in general, the assumptions for linear regression models were met. Furthermore, in each regression model, we controlled for effects of gender, age, and education (see supplementary table 1).

## Results

A linear regression model, with PoS as the predictor, revealed a significant negative relation to SD (*β* = − 0.28, *p* < 0.001, CI_95_ [− 0.36, − 0.20], *R*^2^ = 0.08). The same procedure was repeated with CB as a predictor for SD also identifying a significant negative relation (*β* = − 0.26, *p* < *0.0*01, CI_95_ [− 0.36, − 0.20], *R*^2^ = 0.08). Combining both predictors in a linear regression model SD yielded the best relative fit for a model with additive but no multiplicative terms (see supplementary table 2).

A third linear regression model with PoS as a predictor for CB revealed a significant positive relation (*β* = 0.27, *p* < 0.001, CI_95_ [0.19, 0.34], *R*^2^ = 0.08).

Finally, a significant negative mediation effect of CB in the relation of PoS and SD was identified (*β* = − 0.05, CI_95_ [− 0.08, − 0.03]). For the path model of CB, PoS, and SD distance, see Fig. 1.

## Discussion

This study hypothesized that CB and the PoS to a person with schizophrenia are related to reduced SD. Moreover, increased PoS was expected to be associated with higher CB. The association of PoS and reduced SD was expected to be mediated by higher CB. All postulated hypotheses could be approved pointing out that (1) PoS and CB are important predictors of reduced SD from a person with schizophrenia and (2) CB only partially mediate the association between increased PoS and reduced SD.

Empirical investigations testing the impact of CB on the stigma of schizophrenia often concluded that CB are low as it is difficult to identify with someone who experiences symptoms of schizophrenia [3, 18]. However, Schlier et al. [16] tested the validity of the continuum concept about the existence of schizophrenia revealing that CB about schizophrenia seems to be independent from someone’s individual proneness to experience psychotic symptoms. Reasons why PoS and CB were associated with reduced SD might lie within the process of recategorization of a former outgroup member as an ingroup member which was enabled through identification with aspects of a person apart from psychiatric symptoms (also see [4]). Thereby, the PoS might offer an important explanation why people are generally able to generate CB about schizophrenia.

However, the mediating role of CB in the relation of PoS and SD might point out that people with an increased PoS to a person with schizophrenia have generally higher CB which parallels the first part of the CB item “Basically we are all sometimes like this person” [17]. Still, the relationship between increased PoS and reduced SD is only partially mediated by CB pointing out that besides social perception, information processing around the belief in a continuum of symptoms matters too [15].

Limitations of this study should be mentioned. First, to investigate interrelations of CB, PoS, and stigma, we used unlabeled case-vignettes, while reactions to labeled vignettes or persons might differ. Second, interviews were conducted online, which gives respondents great freedom as to when, where, and how they partake in the study. Hence, attention checks have been inserted in the survey which led together with a high drop-out to an overall exclusion of almost half of all participants. Third, we conducted an observational study yielding for associations and mediation models with no inclusion of an experimental design.

Besides this study, empirical evidence from Violeau et al. [19] suggests that pointing out similarities, more than differences, and increasing continuum beliefs while decreasing categorical beliefs matters for schizophrenia-related stigma reduction. Hence, providing holistic case descriptions including the description of regular living situations, human characteristics (e.g., hobbies, daily routines, working situation), and a change in symptom severity might be a way to increase PoS and CB to people with schizophrenia. Upcoming anti-stigma campaigns should include tasks which foster engagement with case descriptions through endorsing similarities apart from psychiatric symptoms and include continuum beliefs’ intervention to increase the theoretical plausibility of the belief in symptom continuity.

## Supplementary Information

Below is the link to the electronic supplementary material.

Supplementary file1 (DOCX 19 KB)

## Data Availability

Data sharing is not applicable to this article as there are still other manuscripts in progress which analyze variables of the data set used in this article.
