# Supplementary material for: Roles of perception of similarities, continuum beliefs, and social distance toward a person with schizophrenia: a German sample study
Source: Soc Psychiatry Psychiatr Epidemiol. 2023 Jan 28;58(4):681–4. doi: 10.1007/s00127-023-02423-1 (PMC10066069; doi:10.1007/s00127-023-02423-1)
Supplement: Supplementary file 1 — Supplementary file1 (DOCX 19 KB) [file 127_2023_2423_MOESM1_ESM.docx]

Submission for publication to Social Psychiatry and Psychiatric Epidemiology

Title: Roles of perception of similarities, continuum beliefs and social distance towards a person with schizophrenia: A German representative sample study

**Supplementary Material**

**Supplementary table 1**

Socio-demographic characteristics of the population sample.

|  | Total population 2019 (%) | Survey 2022 (%)  (n=760) |
| --- | --- | --- |
| Gender ^a^ |  |  |
| Men | 49.3 | 46.1 |
| Women | 50.7 | 53.6 |
| Divers |  | 0.4 |
| Age ^a^ |  |  |
| 18-24 | 13.5 | 9.5 |
| 25-39 | 18.9 | 17.9 |
| 40-59 | 28.1 | 35.8 |
| 60-64 | 7.0 | 11.0 |
| >64 | 22.0 | 25.9 |
|  |  |  |
| Education ^b^ |  |  |
| Unknown/pupil | 4.0 | 0.5 |
| No schooling completed | 3.5 | 0.8 |
| 9 years of schooling | 28.6 | 12.2 |
| 10 years of schooling | 29.0 | 37.8 |
| 12/13 years of schooling | 33.5 | 48.7 |
|  |  |  |

^a^ Data from the Statistical Office Germany 2019

^b^ Data from the German microzensus 2019 >= only available for participants aged 15 years and above

**Supplementary table 2**

Regression coefficients for Social Distance predicted by Perception of Similarities (PoS), Continuum Beliefs (CB) in separate, additive and multiplicative models.

| ***Model*** | ***β*** | ***CI_95_*** | ***p*** | ***R^2^*** | ***ΔR^2^*** | ***AIC*** |
| --- | --- | --- | --- | --- | --- | --- |
| **PoS** | -0.28 | [-0.35, -0.20] | p < .001 | 0.08 |  | 2102.4 |
| **CB** | -0.26 | [-0.36, - 0.20] | p < .001 | 0.08 | 0 | 2107.9 |
| **PoS + CB** | -0.23  -0.20 | [-0.30, -0.14]  [-0.27, -0.13] | p < .001  p < .001 | 0.12 | 0.04 | 2072.0 |
| **PoS * CB** | -0.26  -0.20  0.06 | [-0.34, -0.18]  [-0.27, -0.13]  [-0.02, 0.14] | p < .001  p < .001  p = .09 | 0.12 | 0 | 2071.1 |

Smaller Akaike information criterion (AIC) indicates higher model quality. AIC differences of two and more indicate significant differences.
